# Cardiolipin drives the catalytic activity of GPX4 on membranes: Insights from the R152H mutant

**DOI:** 10.1016/j.redox.2023.102806

**Published:** 2023-07-03

**Authors:** Antonella Roveri, Flavio Di Giacinto, Monica Rossetto, Giorgio Cozza, Qing Cheng, Giovanni Miotto, Lucio Zennaro, Maria Luisa Di Paolo, Elias S.J. Arnér, Marco De Spirito, Matilde Maiorino, Fulvio Ursini

**Affiliations:** a Department of Molecular Medicine, University of Padova, Italy; b Neuroscience Department, Biophysics Section, Università Cattolica del Sacro Cuore, Rome, Italy; c Division of Biochemistry, Department of Medical Biochemistry and Biophysics, Karolinska Institutet, Stockholm, 17177, Sweden; d Department of Selenoprotein Research and the National Tumor Biology Laboratory, National Institute of Oncology, Budapest, Hungary

**Keywords:** GPX4, Selenium, Sedaghatian-type spondylometaphyseal dysplasia, Cardiolipins

## Abstract

The aim of this study was to examine, in biochemical detail, the functional role of the Arg152 residue in the selenoprotein Glutathione Peroxidase 4 (GPX4), whose mutation to His is involved in Sedaghatian-type Spondylometaphyseal Dysplasia (SSMD). Wild-type and mutated recombinant enzymes with selenopcysteine (Sec) at the active site, were purified and structurally characterized to investigate the impact of the R152H mutation on enzymatic function. The mutation did not affect the peroxidase reaction's catalytic mechanism, and the kinetic parameters were qualitatively similar between the wild-type enzyme and the mutant when mixed micelles and monolamellar liposomes containing phosphatidylcholine and its hydroperoxide derivatives were used as substrate. However, in monolamellar liposomes also containing cardiolipin, which binds to a cationic area near the active site of GPX4, including residue R152, the wild-type enzyme showed a non-canonical dependency of the reaction rate on the concentration of both enzyme and membrane cardiolipin. To explain this oddity, a minimal model was developed encompassing the kinetics of both the enzyme interaction with the membrane and the catalytic peroxidase reaction. Computational fitting of experimental activity recordings showed that the wild-type enzyme was surface-sensing and prone to “positive feedback” in the presence of cardiolipin, indicating a positive cooperativity. This feature was minimal, if any, in the mutant. These findings suggest that GPX4 physiology in cardiolipin containing mitochondria is unique, and emerges as a likely target of the pathological dysfunction in SSMD.

## Highlights

• GPX4 activity on artificial membranes requires docking at the interface.
• The R152 residue and cardiolipin are essential components of high-affinity docking.
• When operating on interface GPX4 has a slower catalytic turnover than in micelles.
• The docking triggers a “positive feedback” effect on activity on the interface.
• The R152H mutation restrains enzyme accumulation at the interface.

## Graphical abstract

**Figure:**
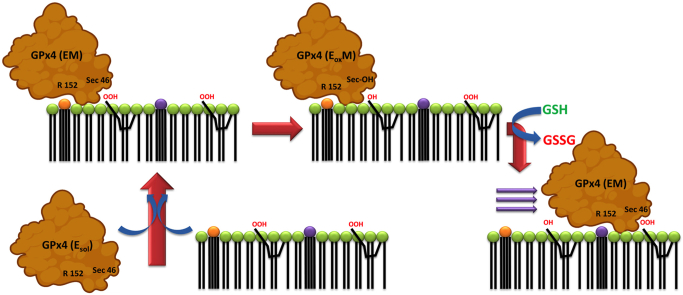

## Abbreviations

BSP: Bromosulfophthalein
SSMD: Sedaghatian-type Spondylometaphyseal Dysplasia
SLPC: 1-stearoyl-2-linoleoyl-*sn*-glycero-3-phosphocholine
SLPCOOH: SLPC-13 linoleoyl hydroperoxide derivative
DOPC: 1,2-dioleoyl-*sn*-glycero-3-phosphocholine
TOCL: 1′,3′-bis[1,2-dioleoyl-*sn*-glycero-3-phospho]-glycerol
TLCL: 1′,3′-bis[1,2-dilinoleoyl-*sn*-glycero-3-phospho]-glycerol
TLCLOOH: TLCL-13 linoleoyl hydroperoxide derivative

## 1 Introduction

The cytosolic Glutathione Peroxidase 4 (EC 1.11.1.12, c-GPX4, from here ahead named GPX4) is the vital selenoenzyme isoform produced by the GPX4 gene [1], which reduces membrane lipid hydroperoxides to corresponding alcohol derivatives using GSH as reducing substrate. This reaction prevents iron-dependent lipid peroxidation of membranes [[1], [2], [3], [4]], which is assumed to lead to a specific form of regulated cell death named ferroptosis [5,6]. Accordingly, missing activity provides a rationale for both the early embryonic lethality of mice carrying a whole gene deletion [7,8] and the specific tissue degeneration produced by conditional knock-out [[9], [10], [11], [12], [13]].

As five out of eight glutathione peroxidase homologs expressed in mammals, GPX4 relies on selenium catalysis. The redox-active Sec46, together with Gln81, Trp136, and Asn137 (GPX4 rat and human numbering), constitute the catalytic tetrad [14]. By proton shuttling these residues support the transient charge separation that provides the nucleophilicity of Sec for the reaction with the hydroperoxide, and primes the cleavage of the O–O bond [15]. Evidence produced using transgenic animals indicates that the Sec of GPX4 is indispensable for its main (essential) biological functions. Few animals carrying a homozygous substitution of the redox-active Sec for Cys, although normally born, develop seizures and degeneration of the parvalbumin-containing neurons in the early days of post-natal life [16].

Proximal to the Sec-containing active site, GPX4 carries a surface exposed cationic area that creates an electrostatic field that is involved in phospholipid polar head binding [17,18]. Docking analysis revealed cardiolipin polar head as the most efficient ligand to GPX4 and the crucial role played by Arg152, interacting with two phosphate groups by a double salt bridge. Surface plasmon resonance (SPR) analysis confirmed the high affinity of cardiolipin for GPX4 [18]. This peculiar affinity for cardiolipin contributed to boosting the debated issue that mitochondria might be the most relevant structure where the reduction of lipid peroxides by GPX4 accounts for vital function in cells. Several additional observations corroborate this notion: i) in GPX4-depleted mice, the reintroduced vital ‘cytosolic’ GPX4 isoform is targeted to mitochondria [7]; ii) peculiar of ferroptosis are dramatic morphological changes of mitochondria, encompassing increased mitochondrial density and reduction of the mitochondrial cristae, despite membrane cell integrity and normal nuclear size [19,20]; iii) presence of mitochondrial damage in the models of GPX4 depletion [11,21]. The notion that ferroptosis primed by GSH depletion or amino acid starvation is facilitated by specific mitochondrial substrates, further provides support for the involvement of mitochondrial aerobic metabolism in producing of lipid peroxidation products triggering ferroptosis [[22], [23], [24]].

In humans, *GPX4* gene defects cause a sporadic, deadly neonatal disease named Sedaghatian -type Spondylometaphyseal Dysplasia (SSMD). SSMD was first described in 1980

as a neonatal condition displaying an unusual pattern of skeletal dysplasia combined with abnormalities of the central nervous system and the heart, which led to mortality due to respiratory failure in the early post-natal days [25]. In 2014 this clinical condition was

associated with the presence of bi-allelic truncating variants of the *GPX4* gene in two unrelated children [26]. The link between defective GPX4 expression and the SSMD diagnosis was further confirmed in 2020 when two additional cases bearing newly described truncations were described [27]. All together the literature reports twenty-five SSMD-affected children since 1980, all dead but three, one female and two males, apparently still alive in 2021 at the age of 6, 11, and 2 years, respectively. These patients share the same missense point mutation in GPX4, i.e., the homozygous substitution of Arg152 with His (GPX4^R152H^), and exhibit only some of the skeletal, nervous, and cardiac abnormalities typical of the deadly forms of SSMD, together with severe additional symptoms [28].

Recently Liu et al. [29] compared both the structure and activity of GPX4^R152H^ mutant and the wild-type enzyme. They show, using recombinant proteins where Cys substitutes for Sec, that the R152H variant exhibits a significantly altered surface around the 152 residue and a destabilized critical loop, both features adding flexibility to the active site by an increased average distance among residues, which leads to weaker interactions. Consistently, enzymatic activity by the conventional GPX4 assay, carried out on extracts of *ad hoc*-transfected eukaryotic cells, resulted in lower activity for the mutated variant.

In the present work, we carried out an enzymological characterization of mutant and the wild-type GPX4, using Sec-containing enzymes produced in an engineered *E. coli* strain suitable for the expression of eukaryotic selenoproteins [30]. Having in mind that GPX4 catalyzes an interfacial reaction on a membrane substrate and that the enzyme-substrate interaction must interplay with a membrane-enzyme interaction at a distinct although proximal site, we break down the kinetic analysis at levels of different complexity: from hydrogen peroxide to phospholipid hydroperoxides in micellar form to monolamellar liposomes. By molecular modeling and computational fitting of the reaction we uncovered the intricate nature of interfacial kinetics where it is cardiolipin that drives the interplay between membrane binding and activity: this function is intriguingly deficient in the R152H mutant.

## 2 Materials and methods

### 2.1 Materials

Avanti Polar Lipids 1-stearoyl-2-linoleoyl-*sn*-glycero-3-phosphocholine (SLPC), 1,2-dioleoyl-*sn*-glycero-3-phosphocholine (DOPC), 1′,3′-bis[1,2-dioleoyl-*sn*-glycero-3-phospho]-glycerol (TOCL), 1′,3′-bis[1,2-dilinoleoyl-*sn*-glycero-3-phospho]-glycerol (TLCL), and polycarbonate filters Whatman® Nuclepore™ Track-Etched Membranes are from Merck, KGaA, Germany; Sep-Pak C18 cartridge are from Waters™ Corp, USA.

### 2.2 Production and purification of recombinant human R152H GPx4 variant (GPX4^R152H^)

The production of recombinant, wild-type human GPX4 (wt GPX4) was previously described [30]. To produce the variant GPX4^R152H^, arginine 152 was point mutated to histidine using the following primer pair:

5′-GATCGGTCTCGTGAAA**CAT**TATGGTCCGATGGAAGAACC

5′-GATCGGTCTCTTTCACAACGCAGCCATTTTTGTCG

directly on the wild-type GPX4 plasmid (pABC2a-HsGPX4). The resulting plasmid (pABC2a-HsGPX4^R152H^) was transformed into *E. coli* C321.ΔA strain. The initial steps of the isolation procedure were carried out in the same manner as described for wild-type GPX4 [30]. The final purification step for enrichment of Sec-containing enzyme [30] was carried out by Bromosulfophthalein-Sepharose affinity chromatography (BSP chromatography), as follows: Buffers: loading, 10 mM KH_2_PO_4_/K_2_HPO_4_, pH 6.3 containing 10% glycerol and 5 mM 2-mercaptoethanol; elution, 10 mM KH_2_PO_4_/K_2_HPO_4_, 200 mM KCl pH 7.5 containing 10% glycerol and 5 mM 2-mercaptoethanol. Sample: 2 mg GPX4^R152H^ diluted 1 to 50 in loading buffer containing 50 mM 2-mercaptoethanol and incubated for 1 h in an ice-cold bath.

Sample was loaded onto the BSP column at 0.4 mL/min; flow rate was increased up to 1 mL/min for the three-bed column volume washing with loading buffer and for elution, which was accomplished by a stepwise gradient with 25%, 50%, 75% and 100% elution buffer.

### 2.3 Mass spectrometry (MS) analyses of purified protein

Homogeneity of GPX4^R152H^ was assessed by LC-MS/MS analysis using a 6520 Q-TOF mass spectrometer controlled by Agilent MassHunter software (B.05.00 version), coupled online with a 1200 series HPLC system through a Chip Cube nano-ESI interface (Agilent Technologies Italia, SpA, Italy) as described previously [30].

### 2.4 Circular dichroism analysis

Sample preparation: purified wt GPX4 and GPX4^R152H^ were buffer exchanged to 25 mM KH_2_PO_4_/K_2_HPO_4_, pH 7.8. Protein concentration was measured at 280 nm and calculated using 30940 M^−1^cm^−1^ extinction coefficient, which was obtained from the ProtParam Tool available on the Expasy Server for the sequence of the cytosolic form of human GPX4 (UniprotKB identifier P36969-2). Protein concentrations used were: 3.5 μM for wt GPX4 and 2.6 μM for GPX4^R152H^. The far-UV CD spectra were collected in a Jasco J810 spectropolarimeter (Jasco Europe Srl, Italy) at 25 °C. Spectra were acquired from 260 to 185 nm in 0.1 cm path length quartz cell, at a scan speed of 20 nm min^−1^ with a response time of 2 s, a bandwidth of 1 nm, and averaged over eight scans. All averaged spectra were corrected by subtracting the corresponding blank (buffer) and then smoothed with a 15 point “adaptative smoothing” procedure. Finally, data were transformed into the mean residue ellipticity, [ζ] (deg cm^2^ dmol^−1^ residue^−1^), according to Kelly [31], using a calculated mean residue weight of 115 Da. The CDSSTR analysis program, available on-line on the Dichroweb site (http://dichroweb.cryst.bbk.ac.uk/html/home.shtml), was used for the analysis of the secondary structure composition. The protein reference set number 6, containing 42 proteins (globular, soluble and denaturized proteins), was used to perform analysis [[32], [33], [34]].

### 2.5 Phospholipid hydroperoxides preparation

SLPC and TLCL were used to prepare phospholipid hydroperoxides containing the hydroperoxyl group at position 13 of linoleic acid (SLPCOOH and TLCLOOH) according to Ref. [35], with minor modifications [36]. Briefly, 1 mM phospholipid suspension in 0.2 M Tris-HCl, pH 8.8 was incubated in the presence of 5 mM sodium deoxycholate and 5000 U/mL soybean lipoxidase, under continuous stirring for 2 h. The mixture (15 mL) was loaded onto a 0.5 mL SepPak C18 cartridge, successively washed with ten volumes of water, 20%, 50%, and 70% (v/v) methanol in water. Finally, phospholipid hydroperoxides were eluted with ten volumes of 80% (v/v) methanol in water and then with the same volume of methanol, brought to dryness in SpeedVac™ (Savant™, ThermoFisher Scientific, Italy), dissolved in 0.5 mL of methanol and enzymatically quantified by GPX4 [36]. Only phospholipid hydroperoxides eluted in 100% methanol were used and total phospholipid content was measured by phosphorus measurement.

### 2.6 Phosphorus measurement

Few microliters (5–10) of phospholipids solutions were dispersed in 1 mL of distilled water and added with 1 mL of concentrated nitric acid (HNO_3_, 68%). The acid dispersions of the phospholipids were then completely mineralized at high temperature by means of a microwave digestion system (UltraWave, Milestone Srl, Italy) using a two-step heating program (25 min from room temperature up to 240 °C, then held 15 min at 240 °C). After mineralization, the solutions were diluted with distilled water to the final volume of 4 mL, filtered (0.45 μm), and analyzed by ICP-OES 5110 VDV (Agilent Technologies Italia, SpA, Italy). The emission lines used were 177.434 nm and 213.618 nm. The P concentration was determined by using a 5-point (0.05–0.1-0.2–0.5–1.0 ppm) calibration curve, obtained from certified single element standard solutions (Agilent Technologies Italia, SpA, Italy).

### 2.7 Liposome preparation

Chloroform solution of SLPC, TOCL (10 mg/mL) and methanol solution of SLPCOOH were mixed at different concentration ratio into a glass tube and brought to dryness by nitrogen flushing. Phospholipids were suspended in 15 mL of 0.1 M KH_2_PO_4_/K_2_HPO_4_, 0.15 M KCl, pH 7.8 at the final concentration of 0.4 mM total phospholipids and 0.08–0.1 mM hydroperoxides. When present, TOCL was 0.08 mM, if not otherwise specified. Liposomes were obtained by extruding lipid suspension 10 times through 0.1 μm Whatman® Nuclepore™ Track-Etched Membrane using a Lipex® Extruder (Transferra Nanoscience Inc, Canada).

### 2.8 Activity of wild-type GPX4 and GPX4^R152H^ on micelles and liposomes

Peroxidase activity was measured at 25 °C in a reaction mixture that contained: 0.1 M KH_2_PO_4_/K_2_HPO_4_, pH 7.8, 2.5 × 10^−3^ M GSH, 5 × 10^−3^ M EDTA, 1.6 × 10^−4^ M NADPH and 0.6 U/mL Glutathione Reductase (EC 1.8.1.7), expressed and purified as described [30]. Triton X-100 (0.1%, v/v) was present in the reaction mixture during activity measurement in the fractions obtained from column chromatography or for kinetic analysis. The reaction was triggered by adding SLPCOOH in methanol.

For activity on liposomes, liposomes (SLPC-SLPCOOH, containing variable amount of TOLC) were diluted to a final KCl and SLPCOOH concentration of 0.075 M and 0.05 mM respectively. The reaction was triggered by adding the enzyme.

Absorbance data at 340 nm were recorded by a Cary UV–Vis multicell Peltier spectrophotometer (Agilent Technologies Italia, SpA, Italy) and enzyme activity was measured as ΔAbs/min during the initial 30–60 s of reaction.

### 2.9 Calculation of the apparent rate constants of the steps of the catalytic cycle

Lipid hydroperoxide substrate was in mixed micellar form with Triton X-100, as described. Enzymes were incubated in 2 mL of the reaction mixture, containing different GSH concentrations (2 × 10^−3^, 3 × 10^−3^, 4 × 10^−3^ M). The reaction was triggered by adding the hydroperoxide substrate (3 × 10^−5^ M). Extinction coefficient of 6220 M^−1^cm^−1^ at 340 nm, was used for calculations; only for H_2_O_2_ the rate of the reaction catalyzed by each enzyme was corrected for the background without enzyme.

Apparent rate constants for GPX4s were calculated by the simplified Dalziel equation [37]:

E/v_0_ = φ_0_ + φ_1_/[ROOH] +φ_2_/[GSH]

where: E is the molar concentration of the enzyme, v_0_ the velocity at different hydroperoxide concentration calculate at different times during the progression curve of the reaction, φ_0_ the reciprocal of the turnover number (typically 0 for selenium-containing glutathione peroxidases); φ_1_ and φ_2_ the Dalziel coefficients, equivalent to the reciprocal of apparent second order rate constant of the oxidative (φ_1_ = 1/*k*_*1*_) and reductive steps (φ_2_ = 1/*k’*_2_) of the reaction, respectively.

### 2.10 SPR analysis of protein-lipid interaction

Liposomes for SPR analysis: chloroform solutions of DOPC or DOPC-TOCL (80-20%) were transferred into a glass tube and chloroform was removed by nitrogen flushing. Phospholipids were suspended in 10 mM Hepes, 150 mM NaCl pH 7.4 at final concentration 1 mM. Liposomes were prepared by extruding lipid suspension as previously described [18]. SPR experiments were carried out at 25 °C with Biacore T100 (Biacore, Sweden) using L1 sensor chip (Cytiva, Sweden) in which lipid bilayers were immobilized. DOPC and DOPC-TOCL liposome solutions were injected in two different flow cells for 25 min at 5 μL/min flow rate; then 10 mM NaOH was flushed for 60 s to remove unbound lipids. For interaction analysis with proteins, wt GPX4 and GPX4^R152H^ solutions from 5 × 10^−8^ to 10^−5^ M in 25 mM KH_2_PO_4_/K_2_HPO_4_, pH 7.8, were injected for 180 s, at 25 °C. After a dissociation period of 300 s 2 M KCl and 50 mM NaOH were used for regeneration.

### 2.11 In silico analysis

The crystal structure of human GPX4 (PDB code: 6HN3) was retrieved from the PDB [38]. All water molecules and ligands were removed; protein was prepared by adding hydrogen atoms using standard geometries using Molecular Operating Environment (MOE) [39]. To minimize contacts between hydrogens, the structure was subjected to FF19SB force field minimization until the root mean square deviation of conjugate gradient was <0.1 kcal mol^−1^ Å^−1^. Protein charges were added using the Protonate 3D tool of the MOE. GPX4^R152H^ was generated using the mutate tool in MOE followed by rotamer explorer. BSP in the phenolic form was modeled, minimized and charged using the MMFF94x force field of MOE. Cardiolipin was prepared as described before [18].

Docking experiments were performed using the MOE Dock program; Triangle Matcher was exploited as the placement method while GBVI/WSA dG as the docking scoring function.

To evaluate the binding affinity of BSP docking poses, XScore was used [40]. X-Score is an empirical scoring functions which estimate the binding affinity of a given protein-ligand complex, including terms accounting for van der Waals interaction, hydrogen bonding, deformation penalty, and hydrophobic effect. The estimated binding affinity is expressed as a dissociation constant of the protein-ligand complex in negative logarithm (pKd).

### 2.12 Reaction rate modeling and computational fitting

The chemical equations used for modeling the kinetics of the enzyme reaction allowed writing a system of 5 differential equations (eqs. (6), (7), (8), (9), (10))), which depend on four parameters (*k*_*A*_*, k*_*B*_*, k*_*1s*_*, k*_*2s*_). This system describes the evolution of the quantities that characterize the whole process and its products, thus allowing a comparison between the model predictions and the experimental data.

We solved the differential equation system numerically using a custom-made script developed in Python and based on the SciPy package.

The workflow consisted of the fit of the experimental curves starting from a set of kinetic parameters (*k*_*A*_*, k*_*B*_*, k*_*1s*_*, k*_*2s*_) and a series of initial conditions for the system variables.

The fit was performed singularly for each experimental curve using the optimized package of the SciPy module, notably implementing the differential evolution algorithm, a well-established stochastic optimization algorithm [41]. As a function to minimize, we considered the root mean square difference between the experimental points and the results of the kinetic models obtained by changing the kinetic parameters.

## 3 Results and discussion

### 3.1 Purification and characterization of recombinant enzymes

**Fig. 1:**
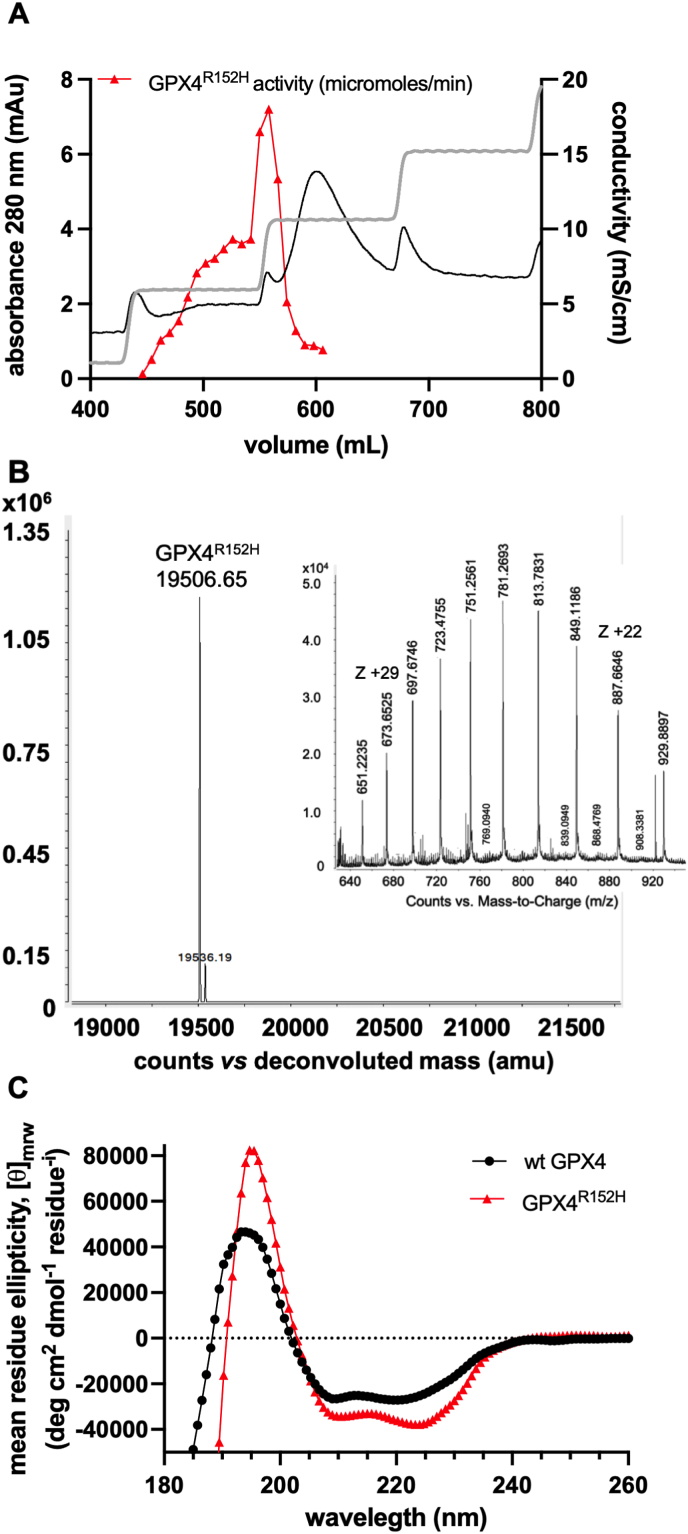
GPX4^R152H^ purification and characterization. A: final purification on BSP affinity chromatography of recombinant Sec GPX4^R152H^ produced in *E. coli*. Absorbance profile at 280 nm shows three main peaks eluting from the affinity column, while GPX4^R152H^ activity is present only in the fractions corresponding to the first peak. B: mass spectrometry analysis of purified GPX4^R152H^. MS spectra and deconvolution obtained from fractions with the highest activity show that GPX4^R152H^ is almost 100% homogeneous; Z +29 and Z +22 indicate the mass with 29 or 22 charges. C: far-UV CD spectra of wt GPX4 and GPX4^R152H^. Recorded spectra reveal a difference in the secondary structure content.

The wt GPX4 and the mutant GPX4^R152H^ were produced using a tailored method with a recombinant *E. coli* strain engineered to code for insertion of Sec at a dedicated UAG codon. As with the wild-type GPX4 [30] also the mutant GPX4, produced by the same procedure, contained up to 80% of inactive protein since Sec was either missed, due to one-codon skipping, or substituted by Lys or Gln. For a final purification step leading to a homogeneous, fully active structurally correct GPX4 protein, we used the BSP-Sepharose affinity chromatography, previously used for the final purification of native [2] and wild-type recombinant GPX4 [30], which proved similarly effective with the R152H mutant (Fig. 1A). GPX4^R152H^ was however bound to BSP at lower pH (6.3 *vs* 7.0) and eluted at lower pH (7.2 *vs* 8.0) and ionic strength (0.1 M *vs* 0.3 M KCl) than the wild-type enzyme [30], indicating a much weaker binding to the stationary phase. This observation is consistent with the critical features of the mutation with respect to the interaction between the protein and the BSP. The ionic binding is supported by the anionic residues of BSP in its phenolic form and cationic residues Lys135 and Arg152 of wt GPX4 [30].

The homogeneity of the isolated R152H protein was verified by MS (Fig. 1B). CD spectra confirmed the presence of a local conformational shift leading to an increase of beta structure in the mutant compared to wild-type GPX4 (Fig. 1C). From the quantitative analysis of the secondary structure content, it was calculated that the mutant has a higher fraction of β-sheets (44% *vs* 28%) and turns (28% *vs* 24%) and a lower α-helix content (5% *vs* 16%) than wt GPX4. This observation agrees with the local conformational shift identified in the crystallographic structure of the Sec-to-Cys substituted variant [29].

**Fig. 2:**
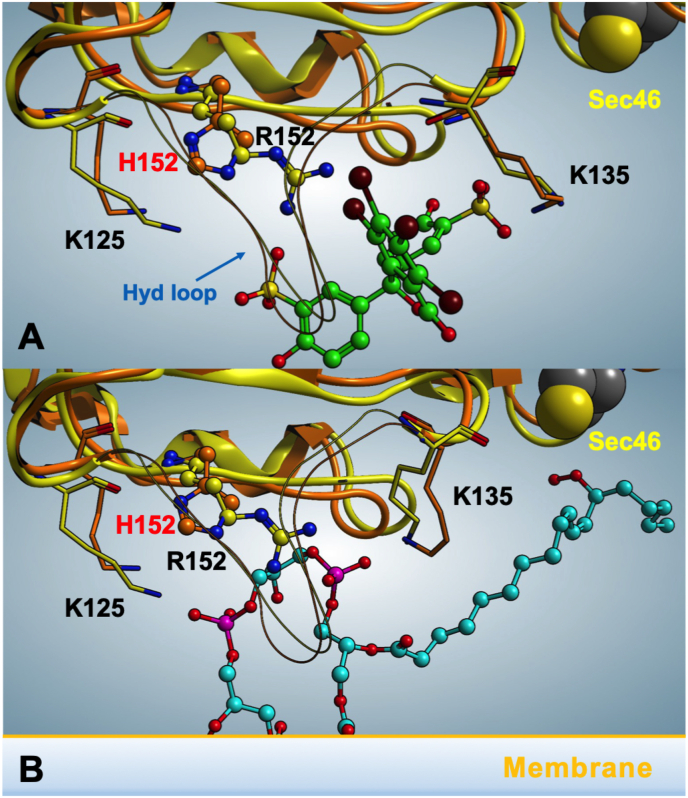
Computational analysis of BSP and cardiolipin polar head interaction with wild-type and GPX4^R152H^. A: molecular docking of BSP (green) against wt GPX4 (yellow)/GPx4^R152H^ (orange). B: molecular docking of cardiolipin (cyan) against wt GPX4 (yellow)/GPx4^R152H^ (orange). The interaction of a hydroperoxide group of cardiolipin floating to the surface and interacting with Sec46 is also reported (see also ref [18]). The most important interacting residues are highlighted. Hyd Loop indicates the hydrophobic loop containing Ile129 and Leu130. (For interpretation of the references to color in this figure legend, the reader is referred to the Web version of this article.)

On the whole, missing Arg152 and the associated structural shift, leading to a decrease of the cationic area on the surface of the mutant GPX4, nicely accounts for the chromatographic behavior on the BSP-Sepharose column. In this regard, the molecular docking of BSP suggests that, while the distance between the Arg152 of wt GPX4 and one of the two sulfur groups of BSP phenolic form is about 2.8 Å, this distance increases up to 4.2 Å in the case of the GPX4^R152H^ (Fig. 2A). This effect is likely due to the significantly smaller size of His as compared to Arg, which lowers its electrostatic contribution. The dissociation constant (*p*Kd, X-Score) estimated in silico is equivalent to 5.87, indicating an affinity of one order of magnitude lower than the previously calculated for wt GP×4 (*p*Kd = 7.21) [30], but still compatible with the binding to the stationary phase of the BSP chromatography. Indeed, the interactions with Lys135 and with the hydrophobic residues Ile129 and Leu130 previously reported for wt GPX4 [30] are conserved in the mutant (Fig. 2 A). Nevertheless, the 6.5 acid constant of His weakens the electrostatic connection between BSP and GPX4^R152H^ at pH 7, contributing to the early elution of GPX4^R152H^.

Of note, the BSP binding region is partially superimposable with the interaction region of the cardiolipin polar head on wt GPX4 [30]. In particular, the region of the protein hosting the sulfuric or phosphoric groups of BSP or cardiolipin, respectively, are conserved and involves the amino acids Lys125, Lys135 and Arg152, as described in Ref. [18].

### 3.2 Wild-type GPX4 and GPX4^R152H^ interaction with phospholipids

**Fig. 3:**
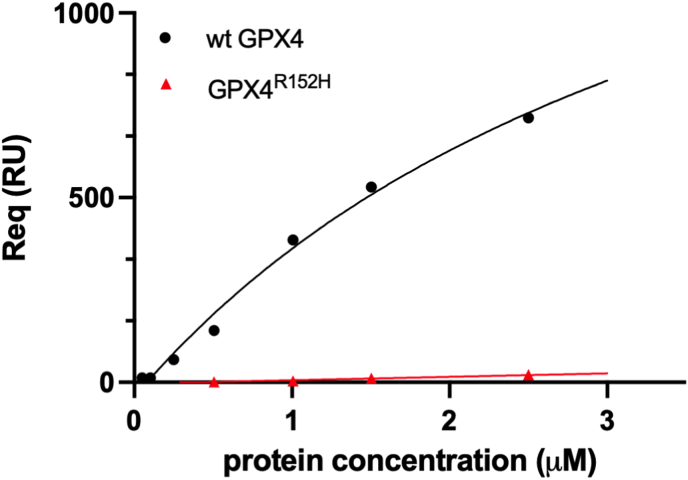
SPR analysis of wild-type or GPX4^R152H^ binding over TOCL and DOPC bilayers. Equilibrium bindings (R_eq_) are measured for various concentrations (5 × 10^−8^ to 2.5 × 10^−6^ M) of wt GPX4 and GPX4^R152H^ on a DOPC and on a DOPC:TOCL (8:2) bilayer in 25 mM phosphate buffer, pH 7.8. R_eq_ values reported in figure are calculated subtracting (R_eq_)_DOPC_ from (R_eq_)_DOPC:TOC_L [18].

The notion that Arg152 is critical for the binding of GPX4 to the polar head of cardiolipin could contribute in unraveling the different catalytic features of the wild-type and the mutant enzyme (Fig. 2B). Arg152 represents the key electrostatic point for the coordination of both cardiolipin phosphates sustained by Lys125 on one side and Lys135 on the other. Therefore, in this simplified scenario, it becomes easy to assume that GPX4^R152H^ loses a fundamental membrane coupling element. Indeed, the distance between Arg152 and the two cardiolipin phosphates is around 2.7 Å in wt GPX4 and increases up to 5.8 Å in the mutant, where His substitutes for Arg, almost abolishing the electrostatic contribution to the binding. Despite this, the interaction of cardiolipin with Lys125 and Lys135, although weaker, is conserved in GPX4^R152H^. This information is consistent with the results obtained from the SPR analysis of the enzyme-cardiolipin interaction. Wild-type GPX4 exhibits a strong interaction with polar head of cardiolipin as previously reported [18], while the mutant displays a minimal interaction only, which makes it difficult to calculate meaningful association and dissociation constants by this analytical approach (Fig. 3).

It is important to note that the binding of GPX4 to the polar head of cardiolipin does not necessarily mean that the enzymatic catalysis will occur exclusively on the hydroperoxides located in the lipid tails of cardiolipin as depicted in Fig. 2B. In fact, consistently with activity measurements (see below), the molecular dynamics [18] complies with the notion that the polar head of cardiolipin serve as docking station for the enzyme allowing it to target hydroperoxides that are exposed on the membrane surface and can access the catalytic redox center.

### 3.3 Activity and kinetic analysis on Triton X-100 micelles

The Specific activity of wt GPX4 and the R152H mutant was measured by the established assay [37] where SLPC-13 hydroperoxide derivative (SLPCOOH) are dispersed in Triton X-100. In six independent preparations of enzymes, wt GPX4 was more active than the mutant: 178.7±32.4 and 34.5±5.2 μmol/min/mg of protein, respectively.

To further investigate the kinetics, we conducted Dalziel analysis on H_2_O_2_, SLPCOOH and TLCL-13 hydroperoxide derivative (TLCLOOH) dispersed in Triton X-100 (Table 1). This procedure permits calculating the apparent rate constant for the oxidative and reductive steps of the ping-pong catalytic cycle. We used H_2_O_2_ to analyze the mechanism of the redox transition at the catalytic center, and SLPCOOH or TLCLOOH to get insight into the role of the structure of phospholipids interacting with the enzyme in a mixed micellar form. The results indicate that the ping-pong catalytic mechanism of GPX4 is conserved in GPX4^R152H^. This is supported by the fact that the apparent rate constants for H_2_O_2_ are the same for both the wild-type and mutant enzyme. Furthermore, the measurements are consistent with the previously reported higher specific activity of the wild-type enzyme on phospholipid hydroperoxides and also pinpoint the role of the structure of the phospholipid bearing the hydroperoxide group. Nevertheless, the information of a lower specific activity, *per se*, hardly accounts for the severe phenotype resulting from the mutation. Yet mixed micelles, while suitable for measuring some aspects of catalysis, are not representative of the constraints of the physiological function of a peroxidase acting on membrane substrates.

**Table 1:** Apparent second order rate constants of human recombinant wt GPX4 and its R152H mutant (GPX4^R152H^) for the oxidative step (*k*_*1*_) and for the cumulative reductive steps (*k’*_*2*_) of the catalytic cycle using different peroxidic substrates. Measurement details under material and methods.

H_2_O_2_	*k*_*1*_ (M^−1^s^−1^)	*k*_*2*_’ (M^−1^s^−1^)
wt GPX4	9.6 × 10^4^	4.0 × 10^3^
GPX4^R152H^	9.4 × 10^4^	2.8 × 10^3^
**SLPCOOH**		
wt GPX4	2.6 × 10^7^	4.4 × 10^4^
GPX4^R152H^	2.5 × 10^6^	2.7 × 10^5^
**TLCLOOH**		
wt GPX4	3.1 × 10^8^	4.0 × 10^4^
GPX4^R152H^	8.4 × 10^7^	5.0 × 10^5^

LEGENDS TO THE FIGURES.

### 3.4 Activity on liposomes

We measured the enzyme's activity, as initial reaction rate at varying molar enzyme concentration, on monolamellar liposomes whose composition and shape were carefully controlled (Supplementary data 1). Indeed, the analysis at increasing enzyme concentrations is the most appropriate for the kinetic analysis of interfacial enzymes [42,43]. To this end, we used liposomes containing the SLPC and the SLPCOOH species (SLPC-SLPCOOH). If not differently specified, when present cardiolipin was 20% of the phospholipid content. Reported here are the results obtained using the TOCL species. We also found that TLCL had the same effects as TOCL (not shown), thus ruling out the relevance of the level of the unsaturation of acyl chains. We also used liposomes containing TLCLOOH. However, although the enzymatic activity was higher, we observed a poor reproducibility of experimental traces, likely attributed to the heterogeneity of the liposome structure. The observed polydispersity (400–1000 nm) of liposomes containing TLCLOOH, indeed, was incompatible with careful interfacial kinetic analysis. For further information on the liposome structure analyzed by light scattering, see Supplementary data 1.

**Fig. 4:**
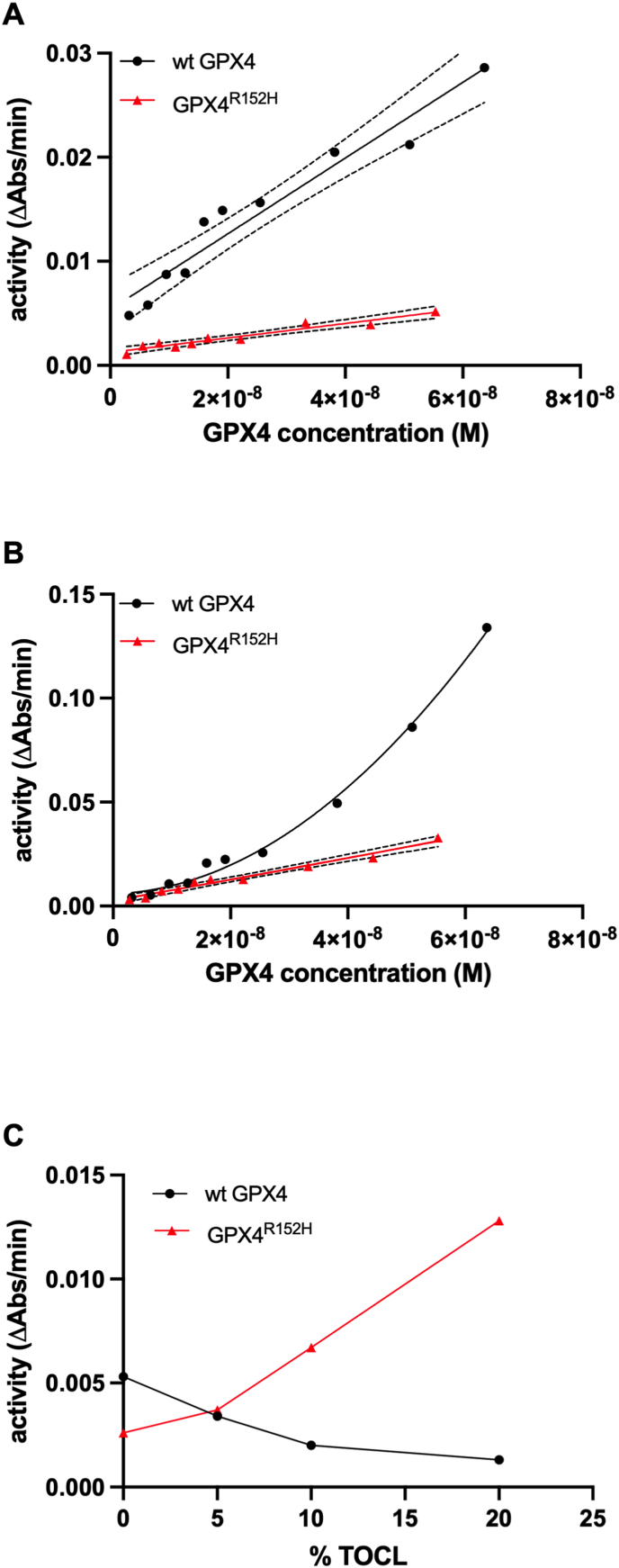
Initial rate of wild-type or GPX4^R152H^ activity on liposomes. Total liposome phospholipid concentration is 0.4 mM and SLPCOOH concentration in the test is 0.05 mM. A and B: increasing concentrations of wild-type GPX4 (3.2 × 10^−9^ M − 6.4 × 10^−8^ M) or GPX4^R152H^ (2.8 × 10^−9^ M − 5.5 × 10^−8^ M). A: SLPC-SLPCOOH liposomes; simple linear regression (continuous line) and 95% confidence intervals (dotted lines) for slopes are reported (R^2^ = 0.9397 for wt GPX4 and R^2^ = 0.9322 for GPX4^R152H^); B: SLPC-SLPCOOH liposomes containing 20% molar fraction of TOCL; simple linear regression (continuous line) and 95% confidence intervals (dotted lines) are reported (R^2^ = 0.9744) for GPX4^R152H^, a second order polynomial fit is reported (R^2^ = 0.9949) for wild-type GPX4. C: SLPC-SLPCOOH liposomes containing Increasing molar fraction of TOCL (0%–20%); the enzyme concentrations for wild-type GPX4 and GPX4^R152H^ are 4.6 × 10^−9^ M and 1.6 × 10^−8^ M, respectively.

Fig. 4A reports the initial rate of the activity on SLPC-SLPCOOH liposomes of both the wild-type and mutant, at varying enzymes concentrations. In these liposomes, the rate of reaction of the wild-type enzyme was higher than that of the mutant. Additionally, both enzymatic activities increased linearly with enzyme concentration. In SLPC-SLPCOOH liposomes containing 20% TOCL (Fig. 4B), a marked increase in activity was observed for both the wild-type and mutant enzymes. Moreover, while the reaction rate increases linearly with GPX4^R152H^ concentration, this was not the case for wt GPX4, where a progressive non-linear activity increase was observed instead.

The whole scenario became even more intriguing when we considered the impact on the reaction rate at relatively low enzyme concentration of increasing the fraction of TOCL in liposomes (Fig. 4C). While the rate of the R152H mutant linearly increased with the fraction of TOCL, a slowing down of the reaction was observed for wt GPX4.

**Fig. 5:**
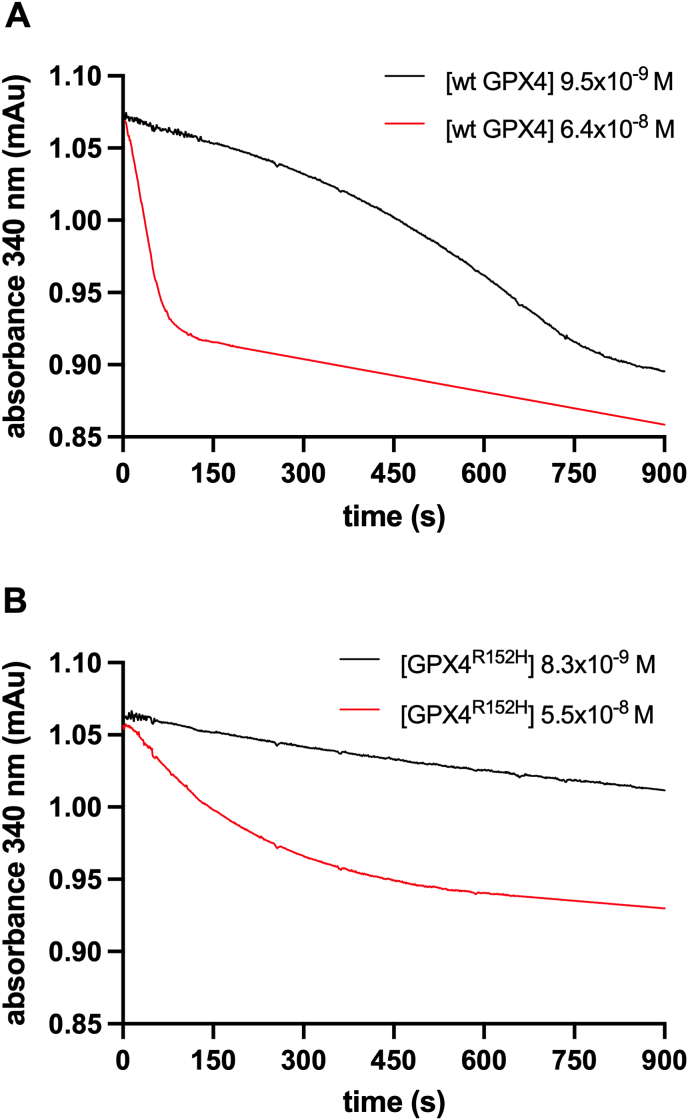
Examples of the progression curves of reaction rates at different concentration of the wild-type GPX4 or the R152H mutant. A: 9.5 × 10^−9^ M and 6.4 × 10^−8^ M wild-type GPX4; B: 8.3 × 10^−9^ M and 5.5 × 10^−9^ M GPX4^R152H^. SLPC-SLPCOOH liposomes contained 20% molar fraction of TOCL.

To get further insight, we decided to analyze, at different enzyme concentrations, the progressive evolution on time of the reaction rate in liposomes containing 20% TOCL. While GPX4^R152H^ produced a monotone decrease in the rate due to hydroperoxide substrate consumption, the reaction rate of wt GPX4 unexpectedly started very low and progressively increased before shifting to the monotone decrease phase. Moreover, this increase disappeared at a higher enzyme concentration. An example of this unusual behavior is shown in Fig. 5. This set of evidence led to developing a working hypothesis that combines the kinetics of lipid hydroperoxide reduction in liposomes with the kinetics of enzyme-membrane interaction at a site distinct from the catalytic redox center. This working hypothesis was assessed by modeling and fitting of the progression curves.

### 3.5 Description of the model and fitting of progression curves

We reasoned that, on liposomes containing SLPC-SLPCOOH only, the kinetics of both, wt GPX4 and the R152H mutant, are close to a diffusion-collision mechanism, where the reaction rate is driven by the availability of hydroperoxide substrate. On the other hand, when liposomes also contain TOCL, the binding of the enzyme to the membranes largely contributes to the reaction rate. The enzyme first binds to TOCL, and then reduces hydroperoxide groups accessible to the redox center. Eventually, the release of the enzyme at the final reductive step of the catalytic cycle [18] drives the shift toward a new binding to a proximal cardiolipin. Further, as a crucial element of our hypothesis, the enzyme does not get released into the bulk solution, but instead remains in close proximity to the membrane, ready for the next binding and series of reactions.

Several solutions, with varying degrees of complexity, have been proposed to describe the kinetics of interfacial reactions in terms of enzyme in bulk solution and membrane associated, substrates and products concentrations. However, some of these models are oversimplified, while others require numerous parameters that cannot be reliably determined by fitting the experimental data. Here, to fit the time course of the peroxidase reaction on membranes, we propose a simple model that integrates the interactions between enzymes and liposomes, with the redox transitions occurring on the two-dimensional space of the membrane surface.

The complete reaction scheme employed to represent the enzyme kinetics is the following:

(1) $$E_{sol}+M\underset{k_{B}}{\overset{k_{A}}{⇄}}\;EM$$

(2) $$EM+SLPCOOH\overset{k_{1s}}{⟶}\;E_{ox}M+SLPCOH$$

(3) $$E_{ox}M+2GSH\overset{k_{2s}}{⟶}EM+GSSG$$

The first reaction (eq. (1)) represents the anchoring of the enzyme, which is modeled as a single-step reaction driven by the association and dissociation rate constants (*k*_*A*_ and *k*_*B*_, respectively) of the reduced form of the enzyme in solution (*E*_*sol*_) to the membrane *(M*, see *infra*). This reaction produces the membrane bound active enzyme (*EM*).

*EM*, operating within a two-dimensional space, reduces lipid hydroperoxides, and is regenerated in the reductive step, which involves releasing and shifting to a different position on the surface. *M* is the molar concentration of all the phospholipids in liposomal form, irrespective of the presence of cardiolipin. The underlying approach examines the collective behavior of an enzymatic activity on the membrane, rather than focusing on a description of individual interactions of specific lipid-enzyme pairs.

The binding process between wt GPX4 or GPX4^R152H^ and *M* was analyzed by integrating information produced by SPR, which not only indicates a much higher affinity of the native enzyme for TOCL than SLPC but also suggests a binding mechanism where the enzyme first binds to the polar head of phospholipid with low affinity and then relaxes to achieve a more stable conformation [18]. Thus, the process was described by two association constants (*k*_*a1*_ and *k*_*a2*_) and two dissociation constants (*k*_*d1*_ and *k*_*d2*_), leading to *EM*. Under the hypothesis of a stationary reaction, the association and dissociation constants of the two different representations can be directly related to each other through the following relations:

(4) $$k_{A}=\frac{k_{a2}}{\frac{k_{d1}}{k_{a1}}+\left[M\right]}$$

(5) $$k_{B}=k_{d2}$$

The second reaction (eq. (2)) is the oxidative step of the catalytic cycle taking place on the membrane surface. It is worth noting that, since the reaction occurs at the interface, the value of the rate constants is different from the kinetic constants calculated by the Dalziel equations on substrates in the micellar phase (Table 1). The physical units of enzyme concentration are also different, as it is described in terms of surface concentration as *EM*.

Finally, the third reaction (eq. (3)) combines the two consecutive bimolecular reactions of the oxidized enzyme with GSH, which are necessary to complete the catalytic cycle. This reaction represents the process required to reduce the enzyme, allowing it to relocate to a different but proximal location within a two-dimensional space.

This modelling approach represents an unavoidable simplification, since neither the spectrophotometric traces nor the SPR data provide sufficient information to independently determine the values of the individual reaction rates. Therefore, we employed a single global constant to reduce the number of free parameters in our kinetic model. It is worth noting that the rate constant *k*_2s_ cannot be directly compared to those calculated using the Dalziel analysis (*k’*_2_, Table 1), as they correspond to two inherently different mechanisms. Nevertheless, by using the steady-state approximation, we can establish a linear relationship between these two values (*k*_*2s*_ *= k’*_*2*_*/[GSH]*) which can be used for a rough comparison. This set of reactions (eqs. (1), (2), (3))), although representing a simplified version of the complex chains of events composing the entire catalytic cycle, effectively captures the most critical features of the enzyme kinetics without the potential issues that may arise with more complex models. These issues, such as overfitting, can occur when a higher number of free parameters are used.

To determine the progression of the reaction and the values of the constants describing the enzyme kinetics, we transformed the reaction equations into a set of differential equations that consider the evolution of the concentration of *EM*, the oxidized substrate concentration, and the production of GSSG. These differential equations incorporated the values of the enzyme in solution [*E*_*sol*_] ,inthe reduced form [*E*_*red*_], which contains the catalytic Sec as Se–H, in the oxidized form, which contains the catalytic Sec as Se–OH, and bound to the membrane [*E*_*ox*_*M*].

(6) $$\frac{d\left[E_{red}\right]}{dt}=-k_{A}\left[E_{sol}\right]\left[M\right]+k_{B}\left[EM\right]$$

(7) $$\frac{d\left[EM\right]}{dt}=k_{A}\left[E_{red}\right]\left[M\right]+k_{2s}\left[E_{ox}M\right]{\left[GSH\right]}^{2}-k_{1s}\left[EM\right]\left[SLPCOOH\right]-k_{B}\left[EM\right]$$

(8) $$\frac{d\left[SLPCOOH\right]}{dt}=-k_{1s}\left[EM\right]\left[SLPCOOH\right]$$

(9) $$\frac{d\left[E_{ox}M\right]}{dt}=k_{1s}\left[EM\right]\left[SLPCOOH\right]-k_{2s}\left[E_{ox}M\right]{\left[GSH\right]}^{2}$$

(10) $$\frac{d\left[GSSG\right]}{dt}=k_{2s}\left[E_{ox}M\right]{\left[GSH\right]}^{2}$$

We numerically solved the system of differential equations (6), (7), (8), (9), (10) using custom-made Python scripts. We started with a different set of kinetic parameters, including *k*_*A*_ and *k*_*B*_ (describing binding constants), *k*_*1s*_, and *k*_*2s*_ (describing rate constants for the different reactions on the two-dimensional surface). We also used initial concentrations of enzymes and repeated the process iteratively. We changed the values of *k*_*A*_, *k*_*B*_, *k*_*1s*_, and *k*_*2s*_ and compared the obtained traces with the experimental data in a fitting process. This led us to obtain a set of meaningful kinetic parameters that minimized the differences with the traces for all the different combinations of liposome composition and enzyme concentration.

### 3.6 Modeling of the time course of the peroxidase reaction

**Fig. 6:**
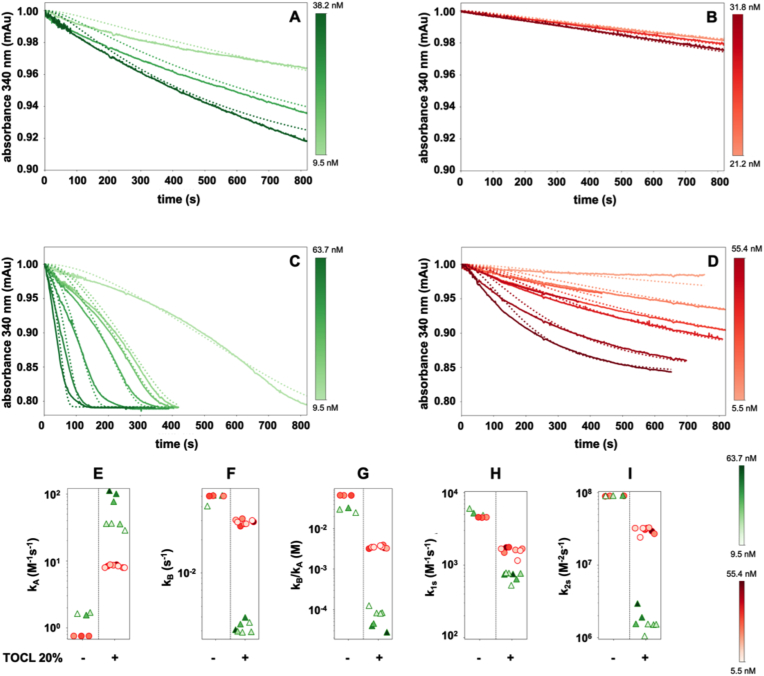
Computational fitting of the progression curves of the reaction. The absorbance (continuous lines) of the peroxidatic reactions on liposomes with the corresponding fit (dotted lines) are reported. Wild-type GPX4 (A) or GPX4^R152H^ (B) were added in a range of concentration (5.1 × 10^−8^ M-6.4 × 10^−9^ M for wild-type GPX4 and 5.5 × 10^−8^ M-2.9 × 10^−9^ M for GPX4^R152H^) to SLPC-SLPCOOH liposomes. In panels C and D the wild-type and the mutant enzymes respectively were used at the same range of concentration on SLPC-SLPCOOH liposomes also containing 20% molar fraction of TOCL. Lower panels (E-F-H-H-I) show the kinetic parameters obtained by fitting of the model with the traces reported in panels A-B-C-D (wild-type GPX4 green triangles, GPX4^R152H^ red dots). Colour gradient indicates increasing enzyme concentration. (For interpretation of the references to color in this figure legend, the reader is referred to the Web version of this article.)

The reaction rates of wt GPX4 and the R152H mutant on liposomes composed of the SLPC and SLPCOOH, reached an equilibrium almost instantaneously and then slowly continued at a rate controlled by the decrease of substrate (continuous lines in Fig. 6A and B). The fitting of the experimental data is consistent with the information obtained by measuring the initial rates (continuous lines in Fig. 4A), demonstrating that the wild-type enzyme is more active than the R152H mutant. Conversely, as mentioned above (Fig. 5), when liposomes also contain TOCL, the traces of wt GPX4 revealed a more intricate depiction of the reaction pathway (continuous lines in Fig. 6C), where the velocity gradually increased before eventually reaching a point of consistent decrease due to substrate consumption. The progressive increase of the rate was less evident at higher enzyme concentrations, when the reaction rapidly reached a maximum and proceeded at a rate mainly controlled by substrate availability. Instead, when GPX4^R152H^ reacted with liposomes containing TOCL, exhibited a monotonic decrease of reaction rate (continuous lines in Fig. 6D). Therefore, for the mutant enzyme, the presence of TOCL only accelerated the process.

The kinetic model was applied to these data, and the dotted lines superimposed on the experimental traces in Fig. 6A–D were thus obtained. The values of the four kinetic parameters that drive the reaction in the kinetic model, shown in Fig. 6E–I, precisely describe the determinants of the time course of the reaction. The parameters *k*_*A*_ and *k*_*B*_ generated by the fitting, show a strong dependence on the enzyme-lipid pairs, changing between wild-type and mutant GPX4. TOCL enhances the affinity between enzymes and liposomes, resulting in higher values for the association constant (*k*_*A*_) and lower values for the dissociation constant (*k*_*B*_). Additionally, the calculated constants differ significantly between the wild-type and mutant enzyme, which is consistent with expectations (Fig. 6E and F). Specifically, in presence of TOCL, the *k*_*A*_ of wt GPX4 for liposomes increases from about 1 M^−1^s^−1^ to 20-100 M^−1^s^−1^ and that of GPX4^R152H^ from 0.8 M^−1^s^−1^ to about 10 M^−1^s^−1^ (Fig. 6E). As expected, the *k*_*A*_ value for liposomes containing TOCL is higher for wt GPX4. Interestingly, this value also increased with the concentration of the enzyme, as indicated by the darker colors in Fig. 6E. Consistent with the higher affinity, the *k*_*B*_ value decreases in the presence of TOCL (Fig. 6F). However, while the mutant enzyme showed a mild decrease from approximately 5∙10^−2^ s^−1^ to 3∙10^−2^ s^−1^, the *k*_*B*_ of wt GPX4 dramatically dropped to around 3∙10^−3^ s^−1^.

The different values of the docking-undocking parameters (*k*_*A*_ and *k*_*B*_) in the case of wt GPX4 acting on liposomes containing TOCL resulted in an increase in the ratio of enzyme bound to the membrane (*EM/E*_*sol*_) available for the catalytic cycle. This ratio is inversely proportional to the *k*_*B*_*/k*_*A*_ ratio (Fig. 6G). By utilizing equation (4) and the data obtained through the fitting of SPR data [18], an estimated value of approximately 40 M^−1^s^−1^ is obtained for the *k*_*A*_ value, which reasonably corresponds to the range of values found in the present study (28.5–111.5 M^-1^s^−1^).

The changes in *k*_*1s*_ and *k*_*2s*_ observed in SLPC-SLPCOOH liposomes *vs* those also containing TOCL can be rationalized as the effects of the altered lipid environment on the orientation of the enzyme on the membrane surface. The decrease of the value of *k*_*1s*_ observed in Fig. 6H suggests that the interaction of the oxidized substrate with the enzymes is less efficient in TOCL containing liposomes. Notably, also the reductive step associated with the shift to a new substrate (*k*_*2s*_), decreased (Fig. 6I). This effect was markedly more pronounced for the wt GPX4 compared with the R152H mutant. The integrated outcome of the interplay between binding and catalytic activity emerges therefore as the most critical feature that accounts for the difference between the wild-type GPX4 enzyme and the R152H mutant.

In summary, the presence of cardiolipin had distinct impacts on the association of the enzymes to the liposomes, leading to a largely different rate of formation of the *EM* in wild-type and mutant enzymes (as indicated by the higher *k*_*A*_ values of wt GPX4 in Fig. 6E), while decreasing in concert the catalytic constants of the reaction cycle (resulting in lower values of *k*_*1s*_ and *k*_*2*s_, Fig. 6H and I).

Moreover, for wt GPX4 only, in the presence of TOCL, increasing concentration of the enzyme leads to a progressive increase of *k*_*A*_ (Fig. 6E) and therefore a non-linear increase of the concentration of *EM*, the species operating the catalytic cycle. Notably, this is in agreement with a seemingly “positive cooperative effect” also observed when analysis was carried out using the initial rate of the reaction (Fig. 4B).

**Fig. 7:**
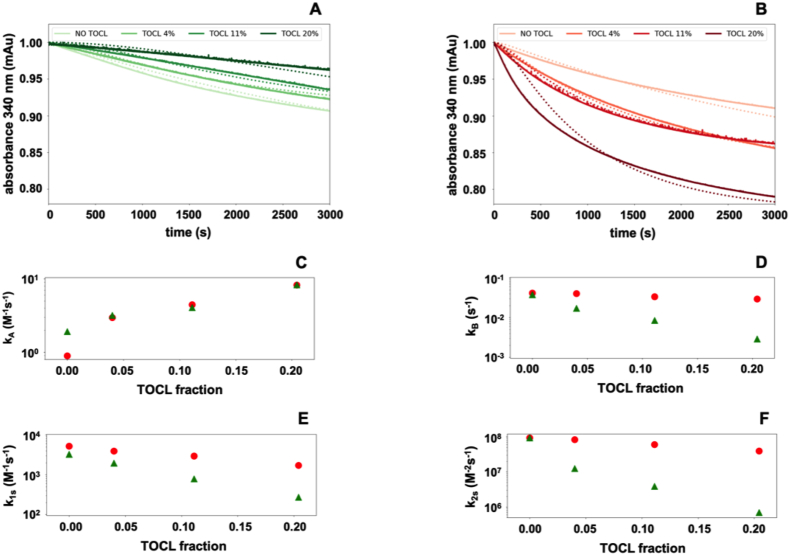
Effect of TOCL on the progression curves of wild-type or GPX4 ^R152H^ activity. Continuous traces indicate the relative absorbance [a.u.], dotted lines the corresponding fit. A: wt GPX4; B: GPX4^R152H^ used at a concentration 4.6 × 10^−9^ M and 1.59 × 10^−8^, respectively. TOCL fractional ratio was - 0, 3, 11 and 20%, and darker color indicates higher fractional ratio. Panels C to F report the kinetic parameters at different TOCL fraction, as obtained from the fit of the traces in the first two panels (wt GPX4 green triangles, GPX4^R152H^red dots). (For interpretation of the references to color in this figure legend, the reader is referred to the Web version of this article.)

Consistent with the notion that the presence of TOCL influences the enzymes' affinity for the membrane, an apparently counterintuitive effect is observed at low enzyme concentrations (Fig. 7A and B). Specifically, the activity of the mutant increases as the concentration of TOCL rises, while that of the wt GPX4 decreases. Notably, the model nicely fits this experimental observation and further provides valuable insights to unravel an apparently contradictory behavior. For both the wt and the mutant enzyme, the *k*_*A*_ value increases with the TOCL fraction, whereas *k*_*B*_, *k*_*1s*_, and *k*_*2s*_ values progressively decrease in the wild-type but not in the mutant enzyme. Additionally, the *k*_*A*_ value obtained for 20% TOCL and low enzyme concentration (Fig. 7C) further strengthen the notion that in the wild-type enzyme *k*_*A*_ increases with enzyme concentration (Fig. 6E).

The dependency of *k*_*A*_ on the enzyme concentration and the TOCL fraction emerges as an obligatory element of the whole mechanism. While on one hand, the oxidative and reductive steps of the reaction are negatively affected by the strong binding between the enzyme and TOCL, the non-linear progressively increasing affinity to membranes (*k*_*B*_/*k*_*A*_) of the wild-type enzyme, collectively leads to increasing catalytic rate by keeping more active enzyme in close proximity to membrane. This accounts for both the paradoxically lower activity of the wild-type enzyme on membranes containing TOCL when at low concentration and for the concentration dependent acceleration of the rate observed in Fig. 4, Fig. 6B. From a mathematical perspective, this would necessitate inserting in our kinetic model a dependence between the value of *k*_*A*_ and the density of enzyme on the surface (*EM*) or adopting a different non-deterministic approach to the modeling of the reaction.

This progressive nonlinear increase in the activity of wt GPX4 at increasing enzyme concentration may be attributed to a “positive feedback” mechanism, which enhances the enzyme-membrane affinity as the enzyme density on the surface increases, as observed in other families of interfacial enzymes involved in signal transduction [44,45]. Notably, this intriguing phenomenon is consistent with our unpublished observation of a specific affinity of rat native GPX4 for recombinant GPX4 bound to the stationary phase of an affinity column. This observation, on the light of the current findings, emerges as particularly significant and sets the course for further specific experimentation.

## 4 Conclusions

Since its discovery, GPX4 has been described as the unique enzyme protecting from lipid peroxidation by reducing to hydroxy derivatives the hydroperoxides of lipids inserted in membranes. Triton dispersion of phosphatidylcholine hydroperoxides is the standardized, most straightforward and suitable substrate to obtain information on specific activity and catalytic mechanism. However, considering the function of GPX4 on membranes, docking and molecular dynamics have revealed two critical elements: the floating of the hydroperoxide groups of phospholipids to the membrane surface, and an enzyme-phospholipid interaction generating a docking site that resides in the cationic area close to the catalytic redox center. The core element of this binding is the interaction between the central phosphate of cardiolipin and the R152 residue of wt GPX4. The existence of an inborn disease produced by the mutation of this residue (GPX4^R152H^) motivated further studies on the role of the R152 residue in the global frame of a deeper analysis of the interaction of the enzyme with the membrane. This mutation is not embryonically or perinatally lethal, although it produces a severe syndrome [28].

The present study addressed the details of the comparison between wt GPX4 and the R152H mutant using, for the first time, homogeneous, active Sec-containing recombinant enzymes. We observed that:

1 The basic catalytic mechanism of the GPX4 peroxidase reaction was not affected by the R152H mutation.
2 Although the higher turnover of the wt GPX4 than the R152H mutant, the kinetic parameters calculated on phospholipid hydroperoxides in mixed micellar form did not differ dramatically, consistently with a similar specific activity.
3 On liposomes containing phosphatidylcholine and its hydroperoxide derivatives, the activity did not differ dramatically between wild-type GPX4 and the R152H mutant, consistently with what was observed in micelles.
4 When liposomes containing cardiolipin, phosphatidylcholine, and its hydroperoxide derivative were used, the time course of the reaction exhibited a progressive increase in reaction rate, dependent on both time and enzyme concentration, in the case of wild-type GPX4. However, his behavior was not observed in the R152H mutant.

To interpret the complex progression curve of the peroxidase reaction in liposomes, we designed a minimal model of the reaction time course encompassing the kinetics of interaction of the enzyme with the membrane and the kinetics of the peroxidase reaction. We converted the set of reactions of the model into a series of differential equations used to calculate the kinetic parameters by fitting the spectrophotometric original experimental traces recording the time course of the reaction. The good fitting of the data revealed that in liposomes:

1 The affinity of GPX4 to the membrane surface is indispensable to forming the complex competent for the reduction of lipid hydroperoxides. This affinity is remarkably much higher for the wt GPX4 than for the R152H mutant, consistent with the cationic nature of the docking site.
2 The higher membrane affinity of wt GPX4 in the presence of cardiolipin, while increasing the docking, decreases the kinetic parameter of the reduction of hydroperoxides, seemingly due to limited mobility on a surface. Thus, the actual reaction rate descends from balancing docking and reactivity. Together, these parameters lead, in the wt GPX4 only, to a time-dependent progressive reaction rate increase due to the progressive accumulation of the enzyme in the active membrane-bound form.

Based on the current evidence, it can be concluded that the R152H mutation minimally affects the peroxidase activity on the membrane surface, unless the membrane contains cardiolipin. However, it is possible that other strongly anionic phospholipids could play a similar role. In the presence of cardiolipin, the wt GPX4, but much less the R152H mutant, accumulates on the surface and reacts with any hydroperoxide present or produced within its radius of action. The dynamics of the release, primed by the regeneration of the reduced form of the enzyme, accounts for the movement of the enzyme over a proximal binding site while remaining in close electrostatic contact with the negatively charged membrane.

The computational data revealed that the wild-type enzyme exhibits a concentration-dependent and cardiolipin-dependent non-linear progressive increase of interaction to the membrane surface (Fig. 6, Fig. 7). This finding is consistent with recent computational studies on interfacial enzymes, indicating the relevance of a “surface sensing” operating a “positive feedback” mechanism that leads to a “cooperative” accumulation of active enzyme on the membrane. These features are believed to be critical for interfacial enzymes in general and particularly when operating in signal transduction pathways [44,45].

Our data validate the notion that mitochondrial membranes containing cardiolipin are a specific site of the GPX4 physiology that when altered in the mutant, emerges as the underlying cause of SSMD. Hence, the latter may represent a novel form of mitochondrial disease. This concept aligns with the recently proposed issue that, in general, impaired mitochondrial function significantly impacts cartilage structure and bone growth among other dysfunctions [46]. These are indeed peculiar features of the specific phenotype of the SSMD. Future, specifically addressed studies are required to validate the proposed connection.

## Declaration of competing interest

The authors declare that they have no competing financial interests or personal relationships that could have influenced the work reported in this paper.

## Data Availability

Data will be made available on request.
