# Supplementary material for: Cardiolipin drives the catalytic activity of GPX4 on membranes: Insights from the R152H mutant
Source: Redox Biol. 2023 Jul 3;64:102806. doi: 10.1016/j.redox.2023.102806 (PMC10345155; doi:10.1016/j.redox.2023.102806)

***Supplementary materials***

*Characterization of liposomes used in activity experiments*

Average size and surface charge were measured by light scattering (DLS) and zeta potential (ζ-potential) analyses performed at 25 °C using a Malvern Zetasizer NanoZS (Malvern Instruments Ltd., U.K.) on fresh liposomes diluted 1:10 in water. Size and polydispersity were determined on the basis of the intensity signal. DLS measurements were performed in triplicate with 10 runs for 10 s measurement.

Average size and surface charge were measured by DLS and zeta potential (ζ-potential) analyses performed at 25 °C using a Malvern Zetasizer NanoZS (Malvern Instruments Ltd., U.K.) on fresh liposomes diluted 1:10 in water. Size and polydispersity were determined on the basis of the intensity signal. DLS measurements were performed in triplicate with 10 runs for 10 s measurement.

|  | size (nm) | s.d. (nm) | polydispersity index | ζ potential (mv) | s.d. (mv) |
| --- | --- | --- | --- | --- | --- |
| SLPC-SLPCOOH | 141.6 | 5.587 | 0.1404 | -1.144 | 0.4914 |
| SLPC-TOCL-SLPCOOH | 133.5 | 3.848 | 0.1046 | -7.223 | 1.919 |
| SLPC-TLCLOOH | 324.6 | 86.04 | 0.46 | -58.72 | 7.336 |

DLS of liposomes of different lipid composition: size and polydispersity. A: SLPC-SLPCOOH liposomes; B: SLPC-TOCL-SLPCOOH liposomes; C: SLPC-TLCLOOH liposomes (TLCLOOH was the 20% of total phospholipids on a molar basis)


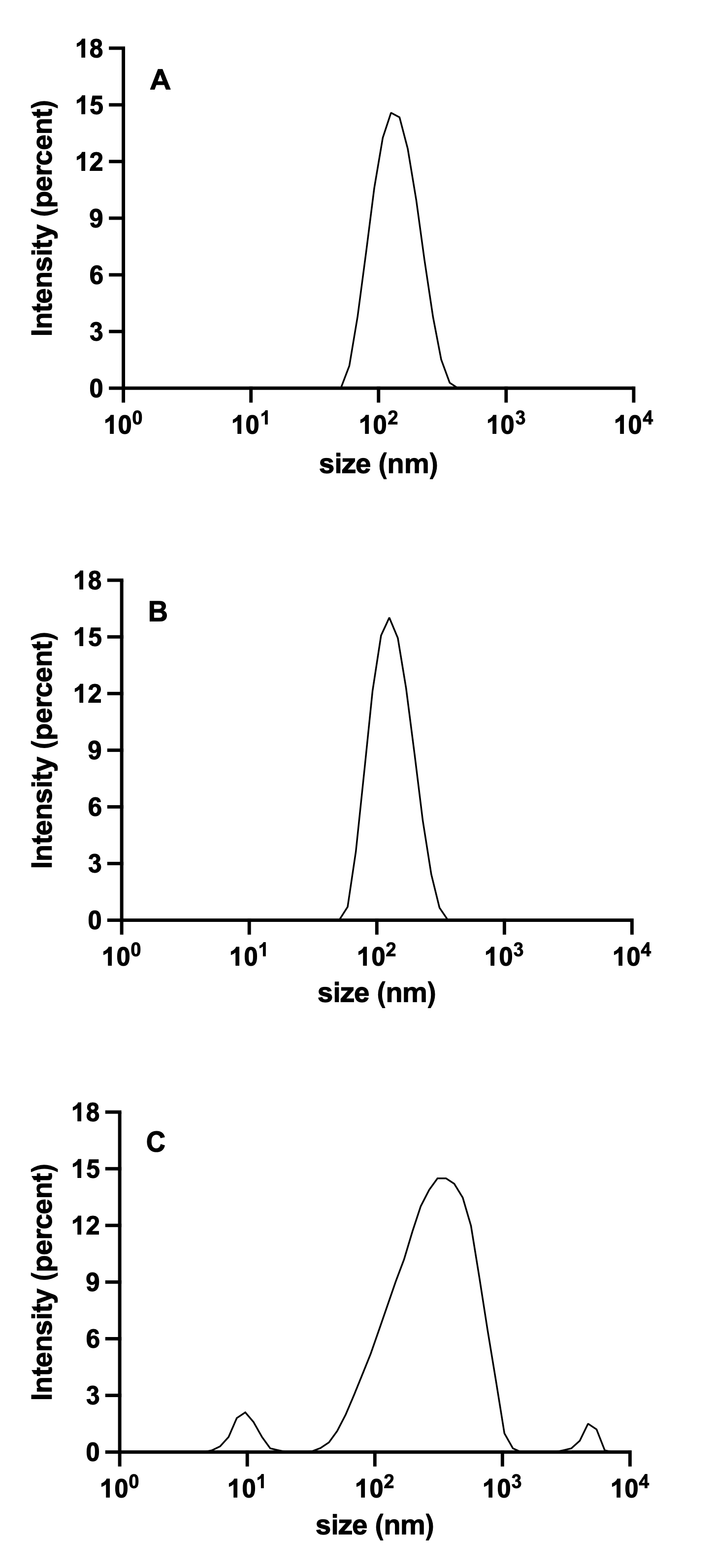

Supplement: Multimedia component 1 [file mmc1.docx]
